# Renewing Medicine’s basic concepts: on ambiguity

**DOI:** 10.1186/s13010-018-0061-4

**Published:** 2018-07-04

**Authors:** Joel Michael Reynolds

**Affiliations:** Rice Family Fellow in Bioethics and the Humanities, The Hastings Center, 21 Malcolm Gordon Road, Garrison, NY 10524-4125 USA

**Keywords:** Ambiguity, Deafness, BIID, Normality, Patient-provider communication, Edmund Pellegrino, Philosophy of medicine, Philosophy of disability, Disability studies

## Abstract

**Background:**

Edmund Pellegrino lamented that the cultural climate of the industrialized West had called the fundamental means and ends of medicine into question, leading him to propose a renewed reflection on medicine’s basic concepts, including health, disease, and illness. My aim in this paper is take up Pellegrino’s call. I argue that in order to usher in this renewal, the concept of ambiguity should take on a guiding role in medical practice, both scientific and clinical. After laying out Pellegrino’s vision, I focus on the concept of normality, arguing that it undergirds modern medicine’s other basic concepts. I draw on critiques by scholars in disability studies that show the concept of normality to be instructively ambiguous. Discussing the cases of Deafness and body integrity identity disorder (BIID), I argue that if medicine is to uphold its epistemic authority and fulfill its melioristic goals, ambiguity should become a central medical concept.

**Methods:**

In this theoretical paper, I consider how central concepts in the philosophy of medicine are challenged by research on experiences of disability. In particular, the idea that medical knowledge produces universal truths is challenged and the importance of historical, cultural, and otherwise situated knowledge is highlighed.

**Results:**

I demonstrate how experiences of disability complicate dominant theories in the philosophy of medicine and why medical practice and the philosophy of medicine should make ambiguity a central concept.

**Conclusions:**

If medical practitioners and philosophers of medicine wish to improve their understanding of the meaning and practice of medicine, they should take seriously the importance and centrality of ambiguity.

## Background

“Medicine qua medicine comes into existence when it appropriates knowledge and skills, no matter what their origin, in order to further its healing purposes.”

—Pellegrino.

“The philosopher is the man who has to cure himself of many sicknesses of the understanding before he can arrive at the notions of the sound human understanding.”

—Wittgenstein.

“May I never see in the patient anything but another creature in pain.”

—Maimonides.

Just 9 years before his death, Pellegrino lamented that our cultural climate had called the fundamental means and ends of medicine into question, leading him to propose a renewed reflection on medicine’s basic concepts, including health, disease, and illness [1]. This call dovetailed with a central theme of his oeuvre, the push for and articulation of a philosophical basis for modern medicine oriented by the realities of clinical practice and human existence [2]. My aim in this paper is take up Pellegrino’s call. I argue that in order to renew the project of medicine today, the concept of ambiguity should take on a central role in “the science and praxis of medicine” [3]. While the philosophy of science has since Kuhn openly wrestled with the historical variability of scientific knowledge and rationality, including the role of sociological factors occasioning its revolutions, biomedical science and practice has on the whole been resistant to admitting the limitations of its paradigms [4, 5]. Given a political atmosphere in which all scientific claims, not just those pertaining to biomedicine, are being called into question, this resistance is especially understandable today. However, I hope to show that this resistance is ultimately misguided.

If, as I argue below, resistance to changes in the understanding of medical phenomena negatively impacts care in multiple respects, then it undermines what Pellegrino holds to be medicine’s fundamental goal: the search for truth in the service of the health and the healing of human beings [3]. After laying out Pellegrino’s vision of medicine as well as the philosophy of medicine, I discuss the concept of normality and the role ambiguity plays—and ought to play—in transforming how we understand the ends of medicine and philosophical inquiry into it. Discussing the cases of Deafness and body integrity identity disorder (BIID), I conclude by arguing that ambiguity should itself become a central concept for medical science and praxis.

### Pellegrino, the philosophy of medicine, and the role of history

Pellegrino defined the philosophy of medicine as consisting in “a critical reflection on the matter of medicine – on the content, method, concepts and presuppositions peculiar to medicine as medicine” [3]. Whether one looks to the titanic promises made by proponents of the Human Genome Project [6] or more recent pledges offered under the auspices of the Precision Medicine Initiative/All of Us Research Program, the gears which churn the medical enterprise forward in the global North too often leverage dogmatic faith in a progressive, promissory vision of medical knowledge and in the infallibility of its guiding concepts [7, 8]. Such a vision stands in stark contrast to the humanistic, patient-centered reflections on the nature and aims of medicine characteristic of doctor-scholars such as Pellegrino and, more recently, Rita Charon, Atul Gawande, and Jay Baruch.

Speaking of his work with David Thomasma, Pellegrino writes, “our philosophy of medicine, and hence the ethics we derive from it, is teleologically structured. It is derived a posteriori from the universal realities of the clinical encounter, i.e., healing, helping, caring, health” [9]. If there is any issue the turbulence of the late twentieth century has brought most forcefully to the realities of the clinical encounter, it is that of normality. Whether with respect to questions of gender, sex, and sexuality, short stature, pregnancy, deafness, racialized medical interventions and categorization, cosmetic surgery, menopause, erectile dysfunction, obesity, ADHD, or any number of mental health issues, the idea that there is a typical or normal human body exhibiting normal behaviors and desires which can serve as a guide for medical praxis has been thrown into question.

Scholars across feminist philosophy, gender, sexuality, and trans studies, critical disability studies, and critical philosophy of race have criticized assumptions about “normality” and how such assumptions feed into common psychological processes, such as implicit bias and confirmation bias, that hamper clinical care and hinder health outcomes [10–16]. By homing in on everything from the selection of clinical research subjects to the treatment of minority groups to the philosophical and historical origins of the concept of “normality” itself, such scholarship has demonstrated that the concept of normality is fundamentally unstable and ambiguous.

It is not surprising that so many debates have turned on the concept of normality, for it is the glue that renders any given modern concept of health, illness, or disease coherent. Just as one must assume or construct a moral exemplar in order to articulate a theory of virtue [17], one must assume or construct a psycho-physiological exemplar in order to articulate a theory of health, illness, and disease. On the whole, these critiques have had a noticeable impact. Despite the influence and standing of scholars like Boorse [18], much contemporary scholarship in the philosophy of medicine follows Tristram Engelhardt and others in holding the concept of health to substantively rely on social, cultural, and historical factors [19]. Notably, even the most trenchant critiques of the concept of normality have not shown it to be entirely useless or incoherent, but problematic and irremediably ambiguous [20, 21]. As philosophers from Wittgenstein to de Beauvoir averred, facing and coming to terms with the ambiguity of life, its forms, and its expressions is one of the more important steps we can take in the project of forging a life worth living [22, 23]. If “the philosophy and ethics of medicine should be grounded in the realities of clinical practice,” as Pellegrino professed, then should not ambiguity itself be a foundation of medicine and its contributions to the good life [9]?

Having laid out the relationship between Pellegrino’s vision of medicine and the philosophy of medicine with critiques of the concept of normality, I will turn to two examples that demonstrate the role and import of ambiguity for a medical praxis grounded in the clinical encounter and steadfastly oriented towards health and healing.

### The ambiguity of normality: deafness

A number of decades ago, the prevailing conceptualization of deafness was in terms of audiological loss [24]. Signing and sign language, though existing in human cultures in various forms since time immemorial, was seen as an unchosen strategy to overcome the inability to hear and one which paled before the advantages of hearing. Fast forward to the present day and thanks to a confluence of sociological, political, and historical factors, one appears uninformed if one fails to contrast this audiological loss view with that of the Deaf (capital D) community. The Deaf community understands ‘deafness’ to refer to a set of rich cultural, historical, and linguistic practices pertaining to groups of people who communicate through signing [25]. While debates both outside and within Deaf communities continue to rage on about things like cochlear implants, the idea that deafness is *solely* and *without argument* defined by audiological loss is today seen as a hangover from a past age, much like understandings of homosexuality as a disease or of certain races or sexes as biologically inferior [26].

Deafness does not demonstrate that the concept of normality—in this case the presence of certain audiological capacities relative to species-level, phenotypic expression—is wrong as much as it shows that the meaning of normality within a given domain and sociopolitical context is often ambiguous and in principle defeasible. Under the sway of normality, we easily lose sight of the fact that much we find clear-cut is not, and much more is up for debate than we realize. In ways that are sadly characteristic of the ivory tower, it took years of testimony, cultural dissemination, and dogged activism for those in the humanities and social sciences to take seriously the fact that Deaf people command and create bodies of knowledge that should indeed count as “evidence.” That is to say, it was in part due to the recognition of these bodies of knowledge *as* knowledge that the reigning biomedical conception of deafness as audiological loss was called into question. Why did this take so long and so much labor?

The answer I propose here, unsurprising to those who have read Foucault and Kuhn alike, is that the “normal science” of medicine in a given epoch is oriented towards stability and clarity based upon extant assumptions concerning its central concepts, principles, and goals. Among other factors, had medical professionals, social scientists, bioethicists, and philosophers been more open to question their assumptions and more attuned to the ambiguity of lived experience, including the experiences of health itself, then it might have taken less of a gargantuan effort for the Deaf community to be recognized on its own terms.

It is important to note that the idea that deafness should be “corrected” does not arise out of evidence that people who are deaf are in pain or suffering. It arises due to the intuition that deafness is abnormal and due to the conviction that medicine’s goals include normalization. Such an intuition, as the twentieth century taught well, is profoundly dangerous when not subject to critical reflection and beholden to the testimony and lived experience of the people to whom the interventions of medicine are aimed and applied [27].

I here argue for the role of ambiguity in medical science and practice because it could work against such dangers, dangers which Anita Silvers argues have been and still can be fatal [20]. Placing the concept of ambiguity at the center of medical praxis would have the additional benefit of giving a greater role to the virtue of humility or, as Eva Kittay puts it, the virtue of epistemic modesty: “know what you don’t know” [28]. The range of human experience is profoundly wide, and there are many sorts of experiences the contours of which we simply can’t imagine thanks to the particularity of the embodied, embedded, and social worlds in which humans live [29].

To understand the practice of medicine as admitting of ambiguity is to admit that that there are many cases where we don’t know, we can’t help, and even with the best laid plans, intentions, and science, we might simply be wrong. As a central concept for medical praxis, ambiguity invokes the need for medical scientists and practitioners to substantively look to other, non-medical ways of knowing for insight—whether it be sociology, history, anthropology, etc.—and look especially to those ways of knowing that are reflexive and critical of reigning paradigms.

### The ambiguity of normality: body integrity identity disorder

BIID is in many respects a more complicated case than d/Deafness. It refers to a very rare condition describing those who feel an intense need to become comparatively impaired, typically through amputation or severance of their spinal cord [30]. It was only after cognitive neuroscientists conducted studies suggesting that it may result from a body-mapping issue related to dysfunction of the right parietal lobe that the dominant medical conceptualization of this condition moved from the psychological (referred to as apotemnophilia) to the physiological [31, 32]. This shift from “abnormal desire” to “abnormal physiology” well exemplifies the role of ambiguity in determining the scope and aims of not just medical intervention, but medical perception of a given phenomenon.

As is unsurprising, the idea of *therapeutic amputation* of a healthy limb or *therapeutic severing* of a healthy spinal cord strikes the majority of practitioners as a contradiction in terms [33]. Indeed, even disability studies scholars have labored to conceptualize the many theoretical and practical issues that BIID raises [34, 35]. Therein lies the rub: it is the confidence over what counts as normal (in this case: being able-bodied and not disabled, having all of one’s limbs, being able to ambulate, etc.) and what constitutes maleficence and beneficence that is the core issue in these debates [36]. Despite neurological evidence suggesting an underlying physiological etiology insufficiently addressed by behavioral therapy and also despite the sociological evidence that people with BIID experience significant and persistent suffering, many practitioners will not carry out these surgeries [37]. This is a case where certainty about the normality of a given condition and the parameters of normal therapy seem to undercut the aims of therapy. This has led some to sadly take matters into their own hands [38].

BIID is a very complex case, and it is quite understandable that the idea of therapeutic amputation would cause some practitioners concern, if not moral distress. Still, perhaps with more sensitivity and comfortability concerning the ambiguity of medical knowledge and of human corporeal variation, the larger aims of the health and healing of human beings would here be better and more capaciously addressed. Whatever position one ultimately takes, both Deafness and BIID demonstrate how the concept of normality is rendered ambiguous when brought under the light of historical and sociological factors central to the production and development of scientific knowledge and the multifactorial conditions by which it progresses.

### In defense of ambiguity

Models of flourishing upon which canonical normative theories are based assume a general minimization of pain and suffering. We may not agree on the *summum bonum*, the greatest good, but we do agree on the *summum malum*, the greatest bad: suffering. A central problem for medicine is the fact that what is taken to be painful or suffered is sometimes not. And that which is taken to be the cause of pain or suffering can turn out instead to be an effect. Whether one looks to debates over Cochlear implants or the revolutions epigenetic research has yielded in molecular biology, the role of psychosocial, historical, and environmental factors continues to force revisions of medicine’s central concepts, both broad (“health”) and narrow (“genetic expression”) in scope.

In The Ethics of Ambiguity, Simone de Beauvoir writes of the human:

At every moment, he can grasp the non-temporal truth of his existence. But between the past which no longer is and the future which is not yet, this moment when he exists is nothing. This privilege, which he alone possesses, of being a sovereign and unique subject amidst a universe of objects, is what he shares with all his fellow [humans]…As long as there have been men and they have lived, they have all felt this tragic ambiguity of their condition...And the ethics which they have proposed to their disciples has always pursued the same goal. It has been a matter of eliminating the ambiguity…Since we do not succeed in fleeing it, let us therefore try to look the truth in the face. Let us try to assume our fundamental ambiguity. It is in the knowledge of the genuine conditions of our life that we must draw our strength to live and our reason for acting [22].

Medical phenomena are multifactorial, scientific knowledge is defeasible, and human wellbeing is equifinal. Each of these truths demonstrate the centrality and import of ambiguity as a description of where we find ourselves and as a condition of how purposive action is ever oriented.

## Conclusions

Pellegrino held that “medicine…is both a science and in many senses one of the humanities” [3]. The concept of ambiguity attunes medical science and praxis, both of which are ultimately in the service of human ends, to better approach the objects and goals of its inquiry and practice. In concert with a Pellegrinian view of medicine as arising out of the realities of the clinical encounter and oriented towards the health and healing of humans, I have argued that medicine ought to reorient itself to become more comfortable with and forthright about the ambiguities that define its objects, bound its goals, and shape its practice both in the lab and the clinic. If the human condition frames medicine and not the other way around, then our age calls not for a more exact, but a humbler medicine.

## Abbreviation

BIID: Body Integrity Identity Disorder
